# Adaptation of a guided low-intensity behavioral activation intervention for people with dementia in Sweden: a qualitative study exploring the needs and preferences of key stakeholders

**DOI:** 10.1186/s12877-023-04606-6

**Published:** 2024-01-30

**Authors:** Oscar Blomberg, Frida Svedin, Paul Farrand, Anders Brantnell, Louise von Essen, Johanna Patriksson Karlsson, Anna Cristina Åberg, Joanne Woodford

**Affiliations:** 1 Healthcare Sciences and e-Health, Department of Women’s and Children’s Health, Uppsala University, Dag Hammarskjölds väg 14B, Uppsala, 751 85 Sweden; 2 Clinical Psychology, Education, Development and Research (CEDAR), Psychology, University of Exeter, Perry Road, EX4 4QG Devon, UK; 3 Industrial Engineering and Management, Department of Civil and Industrial Engineering, Uppsala University, Uppsala, 751 21 Sweden; 4 Department of Medical Science, School of Health and Welfare, Dalarna University, Falun, 791 88 Sweden; 5 Clinical Geriatrics, Department of Public Health and Caring Sciences, Uppsala University, Uppsala, 751 22 Sweden

**Keywords:** Needs, Preferences, Dementia, Psychological Well-being, Intervention development

## Abstract

**Background:**

Despite depression being prevalent in people with dementia, contributing to negative health outcomes and placing increased burden on individuals and family members, access to psychological interventions is limited. A potential solution is guided low-intensity behavioral activation, supported by informal caregivers and guided by healthcare professionals. However, it is necessary to adapt interventions to meet the needs and preferences of key stakeholders to enhance acceptability and relevance. Study objectives were to: (1) explore needs and preferences concerning the content and delivery model of the guided low-intensity behavioral activation intervention; and (2) adapt the intervention to ensure cultural appropriateness, relevancy, and acceptability to people with dementia and their caregivers in Sweden.

**Methods:**

Semi-structured interviews and focus group discussions were conducted with key stakeholders, including healthcare professionals (n = 18), community stakeholders (n = 7), people with dementia (n = 8), and informal caregivers (n = 19). A draft of the written low-intensity behavioral activation intervention and a description of the proposed intervention delivery model were provided to participants. Open-ended questions explored the perceived relevance of the intervention, alongside needs and preferences concerning content and delivery. A manifest content analysis approach was adopted.

**Results:**

Content analysis resulted in three categories: *Content*, *Delivery procedures*, and *Illness trajectory*. Results highlighted a need to consider the intervention *Content* via increased cultural adaptation to the Swedish context, and increasing the inclusiveness of intervention content. *Delivery procedures* were identified as needing to be flexible given the unpredictable nature of caring for people with dementia, with the provision of additional guidance to informal caregivers supporting the intervention. *Illness trajectory* was viewed as essential to consider, with the intervention regarded as suitable for those early in the dementia trajectory, alongside a need to reduce workbook text to minimize burden given dementia symptomology.

**Conclusions:**

The intervention and proposed delivery model were generally well received by all stakeholders. We were able to identify key adaptations to enhance cultural appropriateness, relevancy, and acceptability for a currently neglected population. Results will inform a feasibility study to explore the feasibility and acceptability of the intervention and study procedures to inform the design of a future superiority randomized controlled trial.

**Trial registration/protocol:**

Not applicable.

**Supplementary Information:**

The online version contains supplementary material available at 10.1186/s12877-023-04606-6.

## Background

Approximately 58 million people are living with dementia worldwide, with numbers expected to increase to approximately 153 million by 2050 [1]. Consequently, the prevention and treatment of dementia have become global health and social care priorities [2]. Dementia places a significant burden on people with dementia (PWD), informal caregivers (i.e., persons providing unpaid care or assistance to the PWD, hereafter referred to as caregivers), wider society, and health and social care systems [3]. Depression is common among PWD, with prevalence ranging between 10 and 78% [4, 5], and increases the risk of negative health outcomes, including disengagement from activity [6], mortality [7], poor quality of life [8], and sleep difficulties [9]. Although psychological interventions such as Cognitive Behavioral Therapy (CBT) can be effective for depression in PWD [5], access is limited [10]. Identified barriers include healthcare professionals’ (HCPs) lack of education in psychosocial dementia care [11], exclusion from healthcare services based on diagnosis, high work pressure on HCPs, limited resources, and stigma towards dementia and older adults [12]. This psychological treatment gap is not unique to PWD but represents a global mental health challenge, with only 23% of people with depression in high-income countries receiving at least minimally adequate treatment [13].

Efforts to address the psychological treatment gap include providing psychological interventions via low-intensity CBT (LI-CBT) [14, 15]. LI-CBT techniques are delivered in a self-help format, e.g., via written workbooks, smartphone applications, or the Internet. Guidance from a trained HCP is associated with larger effect sizes than unguided self-help [16]. A promising evidence-based LI-CBT technique for PWD and depression is low-intensity behavioral activation (LI-BA) [17]. Behavioral activation (BA) is an evidence-based depression treatment [18] that seeks to target behavioral avoidance (e.g., disengagement from pleasurable, routine, and self-care activities), a mechanism identified as leading to depression [19]. As well as being symptoms of depression, difficulties initiating and engaging in activities are commonly experienced by PWD due to dementia symptoms [20]. LI-BA uses a simple structured and graded approach that can be used to support PWD re-engage with necessary, routine, and pleasurable activities they have stopped doing [21] and therefore engage in activities PWD and their caregiver associate with well-being. These may include activities such as meaningful participation in family life, continuing usual activities, and finding enjoyment in life [22].

Given the potential of LI-BA for PWD, an intervention [17] was developed in England informed by Phase I (Development) of the Medical Research Council (MRC) complex interventions framework [23]. An innovative aspect of the intervention is that the PWD is supported to use the intervention at home by a caregiver who receives guidance from a trained HCP. Before testing the intervention in the Swedish context there is a need to adapt the intervention alongside key stakeholders (e.g., HCPs, community stakeholders from non-profit dementia and caregiver organizations (hereafter referred to as community stakeholders), PWD, and caregivers). Intervention adaptation is important to increase cultural appropriateness, relevancy, and acceptability for each stakeholder group [24, 25] whilst maintaining fidelity to the evidence-based components of BA [26]. Further, research suggests interventions fail to show similar levels of effectiveness in new settings without contextual adaptation [25], including considering user needs and preferences [27]. A need has also been identified for an increased focus on developing interventions for PWD and depression alongside key stakeholders, including PWD and caregivers, to help improve intervention outcomes [5].

Given the need for intervention adaptation, following Phase I (Development) of the MRC complex interventions framework [23, 24] and informed by ADAPT guidance for adapting complex interventions [28] this study is part of a larger research project [29], with the overall aim of adapting a guided LI-BA intervention for PWD and depression and their caregivers and enhancing implementation potential for the Swedish cultural and healthcare context. Specific objectives of the present study were to: (1) explore the needs and preferences of key stakeholders concerning the content and delivery model of the guided LI-BA intervention; and (2) adapt the intervention to ensure cultural appropriateness, relevancy, and acceptability to PWD and their caregivers in Sweden.

## Methods

### Study design

A qualitative study, placing key stakeholders (e.g., HCPs, community stakeholders, PWD, and caregivers) at the center of the research process [30] and informed by principles from participatory action research [30, 31].

### Researcher characteristics

Authors OB and FS conducted the analysis supervised by ACÅ and JW. OB is a male doctoral student, and FS is a female doctoral student, both have a MSc in Public Health and are trained in qualitative methods. ACÅ is a female professor of Medical Science with a focus on Geriatrics and Implementation Science. JW is a female assistant professor in Healthcare Sciences. JPK is a female project coordinator with a MSc in Public Health. PF is a male professor of Evidence Based Psychological Practice. AB is a male assistant professor in Industrial Engineering and Management. LvE is a female licensed psychologist and professor of Caring Sciences. JW, ACÅ, PF, AB, and LvE are all experienced in qualitative research.

### Participants and setting

Eligible HCPs worked in dementia care (e.g., hospital-based physicians, nurses, occupational therapists), or social care services (e.g., dementia care consultants, caregiver consultants, working in dementia day care services) for PWD or caregivers. Eligible community stakeholders were from relevant non-profit organizations (e.g., patient organizations).

Eligible PWD were: (1) adults with a self-reported diagnosis of dementia (any type); (2) living at home; (3) able to provide informed consent, indicating mild-to-moderate dementia [32]; and (4) able to speak and understand Swedish. Eligible caregivers were: (1) adults self-identifying as caregivers of a PWD with whom they had at least weekly contact; and (2) able to speak, understand, and write in Swedish. PWD and caregivers did not need to be dyadic pairs to be included. Exclusion criteria for PWD and caregivers were: (1) a self-reported diagnosis of a severe and enduring mental health difficulty (e.g., psychosis, type I or II bipolar disorder, and/or personality disorder); (2) visual or auditory impairment that would hinder ability to participate in semi-structured interviews and/or give feedback on the intervention; and/or (3) a self-reported misuse of alcohol or prescription or street drugs reported by the potential participant to interfere with their ability to perform normal activities in daily life.

### Recruitment

HCP and community stakeholder recruitment took place between March and June 2021 across Sweden via non-probability sampling, including convenience [33] and snowball sampling [34], via in-person networks and advertising. A research team member spoke to interested potential participants over the telephone, provided brief verbal information, and sent a study invitation pack by post or e-mail containing a: (1) study invitation letter; (2) study information sheet; (3) reply slip; (4) reasons for non-participation questionnaire; and (5) stamped addressed envelope.

PWD and caregiver recruitment took place between September 2021 and January 2022 across five counties in central Sweden (Stockholm, Södermanland, Uppsala, Västmanland, and Örebro) via memory clinics, social care services (e.g., dementia care consultants, caregiver consultants), and social media (e.g., patient organization and caregiver Facebook groups). Caregivers were also recruited via daycare centers. Recruitment site staff provided brief verbal study information and handed out a study invitation pack to PWD and caregivers.

### Informed consent and eligibility screening

Written informed consent was obtained face-to-face or via post. PWDs were assessed for capacity to provide consent, according to the Swedish Health and Medical Services Act [35]. Thereafter, an eligibility screen was conducted by a research team member. Eligible participants completed a background and sociodemographic characteristics questionnaire with questions including living conditions, occupation, educational background, and self-reported problems with mood or psychological well-being.

### Reasons for non-participation

Informed by previous research [36–38] potential participants who declined participation were asked to complete a reason for non-participation form. Potential participants could provide multiple reasons for non-participation via a closed multiple-choice question and a free text response option. One HCP declined participation due to lack of time (n = 1). Three PWD declined participation due to: being too tired (n = 1); being too stressed (n = 1); having aphasia (n = 1); and not recognizing having dementia (n = 1). Seven caregivers declined participation due to: the PWD having too severe memory impairment (n = 4); PWD not wanting to participate in research (n = 2); too many demands in their own lives (n = 2); lack of time to support the PWD engage with the workbook (n = 1); wanting to use time with the PWD to do things they enjoy (n = 1); the PWD not recognizing their dementia diagnosis (n = 1); and PWD having no problem with well-being (n = 1).

### Intervention

The LI-BA intervention clinical protocol developed in England has been published [17], and is designed for community-dwelling people with mild-to-moderate dementia and depression [21]. A caregiver supports the PWD to gradually re-engage in necessary, routine, and pleasurable activities they used to do, but have stopped doing, and/or identify new activities of similar value, importance, or meaning [21]. Guidance (face-to-face and telephone) is provided to the PWD and caregiver over a 12-week period by an intervention guide (e.g., a HCP trained in the competencies required to guide LI-BA). Supervisors (experts in BA and the intervention), provide training and supervision to intervention guides. A written summary of the intervention delivery model is presented in Additional file 1, and a figure of the intervention delivery model can be found in Additional file 2.

LI-BA techniques are delivered via two written workbooks – one for the PWD to work through the steps of BA and one for the caregiver to help support the PWD work through the steps of BA. Workbooks include therapeutic content following the LI-BA protocol [21], written in a dementia-friendly language (e.g., plain and easy to understand), with illustrations and a case story following an older couple working with the intervention.

### Data collection

Two caregivers dropped out after consent, one due to lack of contact and one due to deteriorated physical health. No participants were found ineligible during screening. A total of 18 HCPs and seven community stakeholders were recruited and interviewed between May 2021 and August 2021, with eight PWD and 19 caregivers between October 2021 and March 2022. Focus group discussions and interviews were recorded with Olympus Digital Voice Recorder WS-853.

#### HCPs and community stakeholders

HCPs (n = 18) participated in either a focus group discussion or individual interview depending on availability and preference. Three focus group discussions were conducted with three, four, and five HCPs, and six participated in individual semi-structured interviews. Community stakeholders (n = 7) participated in semi-structured interviews. Prior to the focus group discussion/semi-structured interview, participants were provided with a written summary of the intervention delivery model developed in England (see Additional file 1) and translated workbook drafts in Swedish. Focus group discussions were facilitated by two research team members (co-authors OB and FS), one as a moderator and one as an observer. The topic guide was partially informed by a cultural adaptation framework concerning treatment delivery elements including delivery format and surface adaptations such as language and illustrations [39] (see Additional file 3). The topic guide was used to explore: (1) first impressions of the intervention; (2) potential professional/non-professional group to support and guide PWD and caregivers using the intervention; (3) preferred intervention delivery setting; (4) type of support and guidance needed to facilitate for PWD and caregivers using the intervention; and (5) perceived relevance of the intervention content and language and potential ways to enhance relevancy, cultural appropriateness, and acceptability. Focus group discussions were held via videoconferencing (Zoom) (n = 3), with semi-structured interviews over the telephone (n = 9), via videoconferencing (Zoom) (n = 3) or face-to-face (n = 1), dependent on preference.

#### People with dementia and caregivers

PWD (n = 8) and caregivers (n = 19) participated in individual semi-structured interviews, provided with a written summary of the intervention delivery model (Additional file 1) and translated workbooks. A topic guide (Additional file 3) explored the same topics as with HCPs and community stakeholders, with additional focus placed on the perceived relevance of intervention content and language and ways to enhance relevancy, cultural appropriateness, and acceptability. Interviews were held face-to-face (n = 19) or via telephone (n = 8), dependent on preference.

### Data processing and analysis

**Fig. 1:**
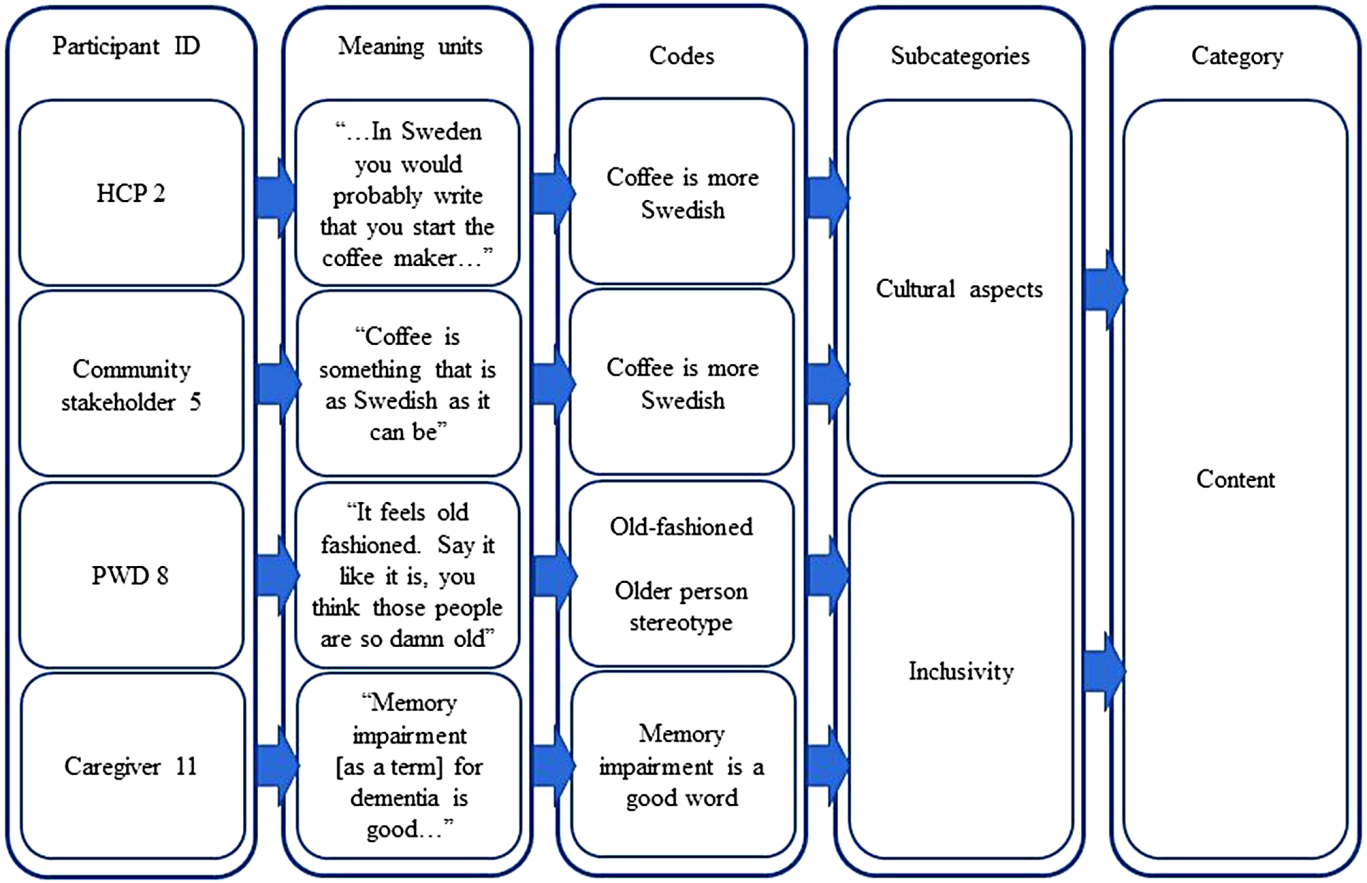
Illustration based on examples from the analysis process

Focus group discussions and individual interviews were transcribed by OB and FS or an external professional transcriber, with transcripts uploaded into NVivo V.12.0 to support data analysis. A manifest content analysis approach was adopted [40] with an illustration of the analysis process provided in Fig. 1. OB and FS read transcripts, coded meaning units using line-by-line inductive coding, and developed a codebook. Each interview was coded by either OB or FS, with co-author JPK coding a sub-set of three interviews to see if further codes would derive from the data. Coding workshops (n = 3) were held with OB, FS, and supervised by co-author ACÅ with variations in coding discussed to develop a deeper and more nuanced understanding of the findings. Codes were categorized into categories, and subcategories by OB and FS. Category workshops (n = 2) were held with OB, FS, and supervised by ACÅ where preliminary categories were discussed and refined. Further, meetings (n = 3) were held with OB, ACÅ, and JW where the data analysis was peer-examined by JW to further establish the credibility and dependability of the analysis. As a qualitative study, we did not explicitly compare and/or contrast the needs and preferences expressed by different stakeholder groups. However, disconfirming cases and divergent opinions are reported to aid understanding and establish credibility [41, 42].

### Sample characteristics

To facilitate the interpretation of supporting quotations, selected characteristics for HCPs and community stakeholders are provided in Table 1 and for PWD and caregivers in Table 2.

**Table 1:** Sample characteristics of HCPs and community stakeholders

Participant ID	Experience working with dementia (years)	Age (years)	Organization type
HCP 1	0–4	25–29	Healthcare
HCP 2	0–4	25–29	Healthcare
HCP 3	0–4	35–39	Social care
HCP 4	0–4	40–44	Healthcare
HCP 5	5–9	40–44	Healthcare
HCP 6	5–9	40–44	Healthcare
HCP 7	5–9	55–59	Social care
HCP 8	10–14	45–49	Health and social care
HCP 9	15–19	40–44	Social care
HCP 10	15–19	40–44	Social care
HCP 11	15–19	40–44	Social care
HCP 12	15–19	45–49	Healthcare
HCP 13	25–29	60–64	Social care
HCP 14	30–34	55–59	Social care
HCP 15	30–34	60–64	Healthcare
HCP 16	40–44	55–59	Social care
HCP 17	40–44	60–64	Social care
HCP 18	45–49	60–64	Healthcare
Community stakeholder 1	5–9	75–79	Local
Community stakeholder 2	10–14	60–64	National community
Community stakeholder 3	10–14	65–69	Local
Community stakeholder 4	10–14	75–79	Local
Community stakeholder 5	15–19	70–74	National
Community stakeholder 6	40–44	70–74	Local
Community stakeholder 7	40–44	70–74	Local

**Table 2:** Sample characteristics of PWD and caregivers

Participant ID	Time since dementia diagnosis (years)	Age (years)	Self-reported problems with mood or psychological well-being	
			< 12 months	> 12 months
PWD 1	Missing	80–84	No	No
PWD 2	Missing	80–84	No	No
PWD 3	< 1	75–79	Yes	No
PWD 4	2	70–74	Yes	Yes
PWD 5	2	80–84	Yes	Yes
PWD 6	2–3	60–64	No	Yes
PWD 7	3	60–64	Yes	Yes
PWD 8	5	75–79	Yes	No
	**Time caregiving for the PWD (years)**	**Age (years)**	**Self-reported problems with mood or psychological well-being**	
			**< 12 months**	**> 12 months**
Caregiver 1	Missing	70–74	Missing	Missing
Caregiver 2	Missing	80–84	No	Yes
Caregiver 3	2	50–54	No	Yes
Caregiver 4	2	60–64	No	No
Caregiver 5	2	75–79	Yes	No
Caregiver 6	2	90–94	Yes	No
Caregiver 7	3	60–64	No	No
Caregiver 8	3	65–69	Yes	No
Caregiver 9	3	65–69	Yes	No
Caregiver 10	3	70–74	Yes	Yes
Caregiver 11	3	80–84	Yes	No
Caregiver 12	4	70–74	Yes	Yes
Caregiver 13	4	75–79	Yes	No
Caregiver 14	4	80–84	No	No
Caregiver 15	5	70–74	No	No
Caregiver 16	6	75–79	No	Yes
Caregiver 17	7	55–58	Yes	No
Caregiver 18	7	75–79	Yes	Yes
Caregiver 19	9	70–74	Yes	Yes

The majority of HCPs were female (94%), with a mean age of 47 and a mean of 17.7 years of experience working with PWD. HCPs worked in healthcare (e.g., as a counselor, nurse, physician, physiotherapist, psychologist, or speech therapist), social care (e.g., as a dementia care consultant, deputy operations manager, caregiver consultant, occupational therapist) or self-employed in dementia care.

The majority of community stakeholders were female (71%), with a mean age of 70, a mean of 19.7 years of experience working with PWD, located across Sweden and worked for national patient organizations (e.g., dementia and Alzheimer’s charities) or local volunteer-led community groups (e.g., local dementia associations).

Fifty percent of PWD were female, with a mean age of 73. All had an Alzheimer’s diagnosis with a mean time since diagnosis of two years. The majority lived with someone (75%) and received support from a caregiver (63%). Six PWD were retired, one was on sick leave, and one worked full-time. The majority (75%) self-reported experiencing problems with their mood or psychological well-being (for example feeling down or sad) prior to the last 12 months and/or during the last 12 months.

The majority of caregivers were female (63%), had a mean age of 72.6, had been in a caregiving role for a mean of 4.1 years and most were retired (68%). The majority (89%) were partners to a PWD and co-resided with them. The majority (74%) self-reported experiencing problems with their mood or psychological well-being (for example feeling down or sad) prior to the last 12 months and/or during the last 12 months.

## Results

Results are presented in two parts, (1) needs and preferences of HCPs, community stakeholders, PWD, and caregivers concerning content and delivery of the guided LI-BA intervention; and (2) subsequent adaptations made to the intervention content and delivery model.

### Needs and preferences

The suggested intervention was well received by all stakeholder groups, with needs for intervention adaptations expressed. Content analysis resulted in three categories: *Content*, *Delivery procedures*, and *Illness trajectory*. See Table 3 for an overview of the categories, subcategories, and disconfirming cases. Category and subcategory descriptions are provided below, alongside supporting quotations, with participant ID and stakeholder type to aid interpretation. Additional supporting quotations are provided in Additional file 4. Disconfirming cases and divergent opinions are reported.

**Table 3:** Categories, subcategories, and disconfirming cases

Categories	Subcategories	Disconfirming cases
Content	Cultural aspects	
	Inclusivity	Neither dementia nor memory impairment are appropriate terms
Delivery procedures	Availability	
	Delivery mode	Lack of belief in telephone support
	Setting	
	Support and guidance	
Illness trajectory	Burden of care	
	Burden of material	
	Timing	Intervention suitable for PWD who have progressed further in their dementia

### Content

Needs and preferences were expressed relating to intervention content, with two subcategories generated.

#### Cultural aspects

All stakeholder groups expressed a need to adapt the workbooks’ case story, illustrations, and language to better reflect Swedish society and the dementia population. Suggestions to increase relevancy included tailoring intervention material to include additional case stories illustrating a variety of different life situations (i.e., a PWD living on their own, or experiencing young-onset dementia) to facilitate PWD and caregivers being better able to identify with the intervention material.

“*Can you have different stories depending on your life situation… for those who live alone? There are too many couples* [in the case stories].” (Community stakeholder 6).

A need for activity examples in the workbooks and case stories, especially for pleasurable activities, that are culturally relevant to the Swedish context, was expressed. For example, a community stakeholder raised that the activity “*taking a cup of tea*” was not culturally relevant and rather: “[taking a] *coffee is something that is as Swedish as it can be*” (Community stakeholder 5). All stakeholder groups expressed the importance of including activity examples in case stories to improve the cultural relevancy of the material, e.g., being out in nature, biking, Nordic walking, and picking mushrooms in the forest, all common Swedish activities.

The quality of the English-to-Swedish translation was raised as important to increase cultural relevancy and appropriateness, with a HCP expressing: “*I can feel in some places that it* [the workbook] *is directly translated from English*.” (HCP 5). However, divergent opinions were expressed concerning the overall language style, with community stakeholders perceiving the language as too academic, complicated, and dense. Conversely, HCPs, caregivers, and PWD considered the text easy to read and understandable. One caregiver considered the language childish and simplistic, thus perceiving the workbook material as derogatory and patronizing to PWD: “*I feel they are speaking to you like a child*.” (Caregiver 9).

#### Inclusivity

Overall, workbooks were perceived as having an old-fashioned and outdated appearance with one HCP describing them as: “*from the 80s.*” (HCP 15). Case stories were perceived as depicting stereotypically older adults, negatively impacting intervention relevancy and potentially exacerbating stigma surrounding dementia and older age. One PWD expressed: “*It* [workbook content] *feels old fashioned. Say it like it is, you think those people* [illustrations depicting PWD] *are so damn old.*” (PWD 8). Some stakeholders considered the names and ages of the couple in the case story to suggest dementia is something only experienced by older people, excluding younger PWD. Case stories were viewed as feeding into stereotypes surrounding dementia and older age and not representing the entire dementia population, potentially preventing intervention engagement:

“*There are no 73-year-olds named Signe these days. 73-year-olds do not have granny clothes or granny hairstyles, they wear jeans and a leather jacket, and the same applies to 90-year-olds, so there is nothing that you can easily identify with here.*” (HCP 15).

A need for caution was expressed in the language used to describe dementia and/or depression. HCPs, PWD, and caregivers expressed different opinions concerning the term ‘dementia’. PWD and caregivers preferred terms such as ‘memory impairment’, perceiving the term dementia as derogatory, embarrassing, and negative, with one PWD explaining:

“[Memory impairment] *is a good word. Everyone understands it, and it is not that dramatic either. If you say dementia, then you directly think of old grandma lying in a bed at home.*” (PWD 3).

There were two disconfirming cases from caregivers who considered neither dementia nor memory impairment to be appropriate terms, with one caregiver stating: “*Memory impairment can be very offensive. You need to come up with something else. Not dementia and not memory impairment, find something else*.” (Caregiver 19). Conversely, HCPs preferred the term dementia or, ‘cognitive disease’ or ‘cognitive impairment’, expressing concerns ‘memory impairment’ would exclude PWD without memory problems.

Divergent opinions concerning the term ‘depression’ were also expressed, with HCPs perceiving the term as stigmatizing, preferring the term ‘low mood’.

“*Not depressed. You can say a bit low, you can have low mood absolutely. But depressed, no, that is a bit too far… Low mood, I can probably agree with.*” (HCP 10).

However, caregivers considered ‘depression’ an acceptable word that is commonly used in everyday language in Swedish to describe feeling low.

### Delivery procedures

Needs and preferences were expressed relating to intervention delivery procedures, with four subcategories generated.

#### Availability

Caregivers and HCPs expressed a need for on-demand, easily accessible, and flexible guidance for caregivers given the unpredictable nature of providing care to PWD. The unpredictability of living with dementia was raised as potentially causing problems with engagement, with a need for flexible on-demand guidance to problem-solve unpredictable challenges that may arise:

“*If they* [the PWD and caregiver] *get stuck then they can make contact* [with the intervention guide] *themselves*.” (HCP 18).

Conversely, PWD preferred regularly scheduled weekly guidance sessions to facilitate keeping track of guidance sessions. Whilst preferences for on-demand guidance were expressed by caregivers and HCPs, the structure of weekly guidance sessions was recognized as an important motivator for PWD and caregivers to “*keep up the pace*” (Caregiver 4) when working through the intervention.

#### Delivery mode

Preferences were expressed by all stakeholder groups for guidance to be provided face-to-face, especially initially, to facilitate communication and understanding, and to build a trusting relationship:

“*Physical meetings can never be replaced as I see it. I have been teaching a lot, and I have had a lot of conference calls, and it works just fine with people you know, but not with strangers. First, it* [a relationship] *has to be established, get to know who is included personally. Then you can use phone or video and things like this, but you have to establish some kind of physical contact first.*” (Caregiver 16).

“*I think it is somehow easier, or at least I think it is easier to explain to caregivers with the help of gestures and pictures. You can draw and you can explain in a completely different way than you can over the phone. I think there will be a lot of misunderstandings. I think it is much better to meet physically*.” (HCP 9).

Whilst initial face-to-face meetings were preferred to facilitate establishing a relationship with the intervention guide, subsequent telephone guidance was expressed by HCPs and caregivers as a simple and convenient way to solve problems experienced with the intervention.

A preference for printed workbooks, rather than digital solutions was also expressed, with one caregiver stating: “*I am old-fashioned*, [I want] *to be able to hold the material* [in my hands].” (Caregiver 3). It was considered especially important that the PWD workbook was in print form, whereas providing the caregiver workbook in digital form was considered by some HCPs and caregivers as a way to enhance ease of access. It was however acknowledged that future generations of PWD might prefer digital solutions:

“*Our patient group* [PWD] *today, they are perhaps more used to being able to have it* [workbook] *on paper and may not be so digitally savvy* [to have a digital workbook]. *But this* [intervention] *is not something we are starting today but something we will start within a few years, perhaps by then our patient group is also younger and more computer-savvy* [and can have a digital workbook].” (HCP 1).

#### Setting

Overall, stakeholders described key characteristics of face-to-face guidance sessions as located somewhere convenient, familiar, private, and safe, with a need for flexibility voiced concerning the setting. Opinions concerning which intervention settings meet these key characteristics were diverse across all stakeholder groups and ambiguous within each stakeholder group. All stakeholder groups described the home as a suitable environment. However, within all stakeholder groups barriers to home delivery were also mentioned. For example, HCPs expressed caregivers may not be able to speak freely about the PWD in their home, and one community stakeholder described a need for caregivers to get a break from their home environment. HCPs expressed some PWD and caregivers may not want others in their home. Healthcare settings for guidance sessions were described by community stakeholders and some caregivers as both convenient and familiar. However, HCPs, other caregivers, and PWD expressed PWD and caregivers may be tired of healthcare settings:

“*It depends on the people in question. The easiest thing is if you meet at home, that is the easiest thing for the people involved. You do not have to go anywhere. We have probably had enough of hospitals. He* [the PWD] *does not want to go there* [to hospital] *unnecessarily*.” (Caregiver 6).

#### Support and guidance

The importance of providing additional training and guidance to caregivers e.g., intervention-specific training, dementia education, and additional ‘booster’ guidance sessions was expressed:

“*The caregiver probably needs* [training] *at the beginning, to get an understanding of the* [dementia] *disorder, the different dementias that exist, how to deal with it, what can you expect, and remind that not everyone follows this* [dementia progression] *exactly, but there are variations as much as there are different individuals.*” (Caregiver 19).

Experience-based knowledge of dementia and the caregiver role was considered essential for the intervention guide, rather than needing to have a specific healthcare profession:

“*Actually, someone who has had previous experience from being a caregiver, who has been personally involved … having seen it and maybe followed the disorder* [dementia] *from the first time when someone forgets to bring their hat until you do not know your name anymore.*” (PWD 3).

However, some health and social care professional roles considered suitable to provide intervention guidance included dementia care consultants (dementia support teams who provide community-dwelling PWD and their caregivers with general guidance and advice on communication and treatment), occupational therapists, and nurses:

“*Occupational therapists* [are suitable]. *I have worked a lot with occupational therapists when we have developed dementia care, and there is that foundation, that knowledge. They are good at finding things that support, help, aids… They have the foundation they can develop further*.” (Community stakeholder 6).

### Illness trajectory

Needs and preferences were expressed relating to needing to consider the dementia illness trajectory to facilitate engagement, with three subcategories generated.

#### Burden of care

An overall need to minimize the treatment burden for those receiving and supporting the intervention was expressed. Concerns were raised regarding the treatment burden placed on caregivers to support the PWD beyond their everyday caring activities:

“*There are great demands placed on caregivers at the moment. It will probably become more and more. If the PWD cannot keep his diary himself, fill it in himself, think for himself, and get it done, then you* [the caregiver] *have to do the thing this person cannot handle. So, the caregivers have to take over this. And I would never have put myself up to it, because I had such a hard time anyway.*” (Caregiver 19).

#### Burden of material

Whilst the workbook material was perceived as good and important, all stakeholder groups were concerned about the quantity of workbook text. *“There was a lot of text* [in the workbook].*”* (PWD 6). Concerns that workbook text would overwhelm caregivers when trying to read and understand the materials, as well as subsequent difficulties PWD may experience reading, understanding, and remembering were expressed. A need was voiced to reduce redundant text to enhance simplicity and understanding, and for the material to adopt a more “step-by-step” approach to minimize burden and reduce potential feelings of being overwhelmed.

#### Timing

Overall, stakeholders considered the intervention most suitable for PWD early in the dementia trajectory, with suggestions that the intervention could become part of the care plan for the PWD and caregiver at diagnosis:

“*I do not think this* [intervention] *is applicable if you are too advanced in the illness. This must be put in place immediately upon diagnosis, because then the person is in an early stage and then something like this can be a help, as part of, rehab is maybe the wrong word but as part of a care plan.*” (Community stakeholder 2).

Provision of the intervention early in the dementia trajectory was perceived as facilitating engagement with the material. One PWD stated: “*This* [intervention] *applies to early support, while you are still able to read and analyze.*” (PWD 7), and others suggested the intervention could be provided when dementia symptoms are first noticed. However, one caregiver considered the intervention only to be relevant later in the progression of dementia:

“*For those who have come further* [in the progression of dementia] *it is probably very good.* [But] *not for me yet, no.”* (Caregiver 7).

### Adaptations made to the intervention content and delivery model

**Fig. 2:**
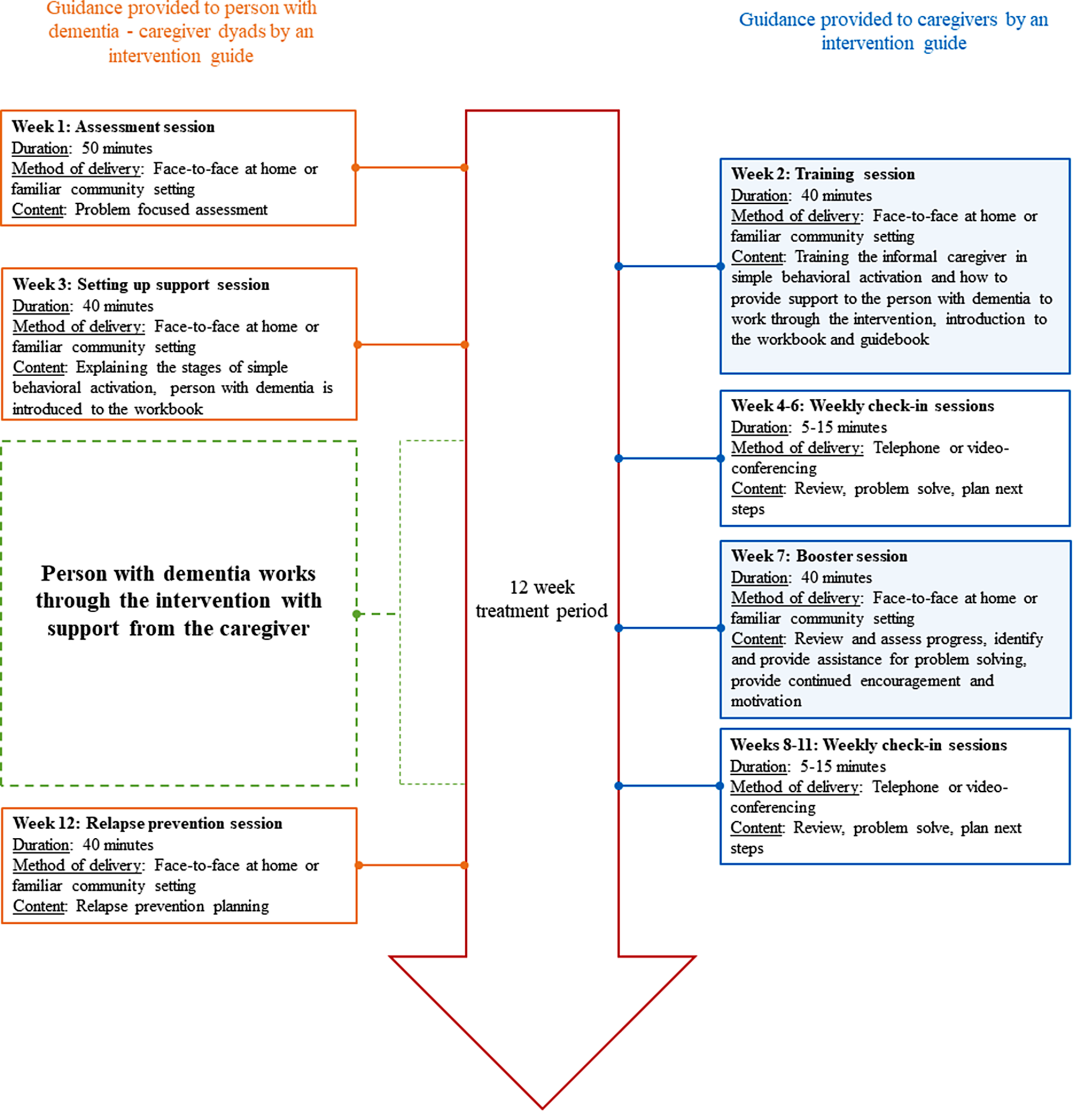
Proposed intervention delivery model. The shaded blue boxes indicate the additional guidance sessions added for caregivers.

To enhance the cultural appropriateness, relevancy, and acceptability of the intervention, findings were used to inform adaptations to the intervention content (i.e., language and illustrations) and procedures to inform the intervention delivery model (i.e., additional support for caregivers). Examples of adaptations made to the intervention material and delivery model are provided in Table 4 and the proposed intervention delivery model is illustrated in Fig. 2.

**Table 4:** Examples of adaptations of intervention content and delivery model

Element of intervention material and delivery model	Needs/preferences	Adaptation
**Content**		
Style	Workbooks are described as old-fashioned	A professional design company redesigned the workbooks and developed new modern illustrations
Language	Text directly translated from English to Swedish	Text re-written by native Swedish speakers
Case stories	The case story is stereotypical and not representative of the varied life situations of PWD	Three new case stories developed representing one older married couple, one single-living PWD, and one person with young-onset dementia with a partner and young children. Variations in names, age, ethnic background, and gender
Examples and illustrations of activities	Activity examples used in the case story and the wider workbook are not relevant to the Swedish context	Activity examples and illustrations that are relevant to the Swedish context added, for example, taking “fika” (coffee), and Nordic walking
Illustrations of PWD and caregivers	Illustrations of PWD and caregivers were stereotypically older adults	Illustrations of PWD and caregivers of different ages, genders, and ethnic backgrounds developed
Information about dementia	Additional education about dementia is needed for caregivers	Information about common dementia types and symptoms added to the caregiver workbook
**Delivery procedures**		
Delivery model	A need for additional caregiver training to provide support to the PWD	A separate caregiver training session with the intervention guide added
Delivery model	A need for additional face-to-face guidance sessions for the caregiver	A face-to-face (or video-conference) guidance session mid-intervention for the caregiver (booster session) in addition to weekly check-in support
Delivery model	Preference for face-to-face guidance versus the telephone	An option for video-conferencing guidance sessions added to potentially mirror face-to-face guidance
**Illness trajectory**		
Burden of material	The amount of text was overwhelming	Redundant text reduced and language simplified

## Discussion

Overall, the idea of a LI-BA intervention for PWD, supported by caregivers and guided by a HCP, was well received. Three categories were identified *Content*, *Delivery procedures*, and *Illness trajectory.* Findings related to intervention *Content*, highlighted a need to consider increased cultural adaptation to the current Swedish context and increase inclusiveness to better represent PWD in a variety of different life situations. Related to *Delivery procedures*, a need was identified for the provision of additional and flexible guidance to caregivers supporting the intervention. Concerning *Illness trajectory*, preferences were expressed for providing the intervention early in the dementia trajectory, alongside a need for a simplified intervention to reduce the burden placed on both caregivers and PWD. Findings were used to inform adjustments to workbook case stories, illustrations, and language as well as for improvement of the suggested intervention delivery model.

Findings indicated a need to adapt a number of peripheral [43] intervention components (i.e., engagement and treatment delivery components and language) to improve the cultural appropriateness, relevancy, and acceptability of the intervention. Results correspond to findings reported in a recent systematic review that highlighted a need to tailor intervention material content and intervention delivery to improve the engagement of PWD and caregivers in dyadic non-pharmacological interventions [44]. Our results are also in accordance with a person-centered approach to dementia care [45], placing importance on involving PWD and caregivers in care planning and decision-making, as well as providing a sense of choice and control [46].

Findings also suggest access to an intervention guide with knowledge and experience of caregiving and dementia and the provision of face-to-face guidance sessions as important aspects to build a trusting care relationship. This is consistent with wider research highlighting the importance of accessing an intervention guide, who is caring, knowledgeable, and competent, to enhance engagement within dyadic non-pharmacological interventions for PWD and caregivers [44]. Preferences for face-to-face support found in the current study may be based on perceptions that interventions traditionally provided in person cannot be delivered remotely. However, this may represent a barrier to future intervention use, since the intervention delivery model includes both face-to-face and remote guidance (via telephone and videoconference). Potential solutions are to provide training for PWD and caregivers in initial face-to-face guidance sessions to increase their technological literacy and access to technology use [47].

A relatively high degree of need was expressed for enhancing the cultural appropriateness of the intervention, such as the inclusion of activities in case stories that better represent Swedish culture was identified. Cultural adaptations are commonly expected, for example when adapting interventions developed originally for Western cultures for local populations in non-Western cultures [48, 49]. However, to the best of our knowledge, there has been less focus on the cultural adaptation of interventions developed in what may be perceived as relatively similar high-income Northern European settings (i.e., the UK and Sweden). The need for cultural adaptation also included intervention modernization via new illustrations and adapting intervention content and case stories to reflect wider demographic and societal changes e.g., representing those who are unmarried/divorced, without children, the geographic spread of families [50, 51], and people with young onset dementia and their families [52]. Consequently, two additional case stories depicting PWD and caregivers in different life situations and modern illustrations were developed. A related finding was a reported need to consider providing the intervention in digital form in the future. Given the digitalization of society, some stakeholders may prefer a digital intervention, indicating a need for future intervention adaptation to follow societal developments [53]. Preferences for digital healthcare interventions have been expressed by people with young-onset dementia and their partners [54]. Further, given an increasing number of caregivers are “distance caregivers” [50] there is an increasing need to develop sustainable dyadic interventions provided digitally [55].

Adjustments to workbooks to ensure inclusive and non-stigmatizing language and illustrations included not using the term ‘dementia’ which some stakeholders perceived as stigmatizing, with PWD and caregivers preferring the term ‘memory impairment’, similar to a previous study [56]. The depiction of PWD and caregivers in illustrations and the case story as older adults were also perceived as potentially stigmatizing and failed to represent the wider dementia population. Common stigmatizing attitudes held by both members of the public and HCPs include the belief dementia is part of the normal aging process and that discrimination towards PWD has been found to increase with the person’s age [57]. Importantly, dementia stigma has been found to prevent PWD from seeking diagnosis as well as information and support [58]. Lack of acceptance regarding the term ‘dementia’ among PWD and their caregivers may indicate a wider need for the development of evidence-based stigma reduction interventions [58]. Whilst the preferred term ‘memory impairment’ was adopted in the intervention, it may overlook and impact the acceptability of the intervention for individuals with non-Alzheimer dementias, whereby cognitive impairments other than memory loss may be more pronounced.

Consistent with the proposed delivery model, PWD expressed preferences for structured guidance at the same time on a weekly basis, whereas caregivers preferred flexible on-demand guidance. Caregiver preference for flexible intervention delivery has been identified elsewhere [56], and caregivers of PWD have emphasized factors such as fluctuations in motivation and mood, lack of time, and the need to prioritize other tasks as barriers to intervention engagement [59]. Importantly, incongruent healthcare preferences between PWD and their caregivers have been found to predict greater relationship strain and worse mood in the PWD [60]. Incongruent intervention preferences expressed in the present study suggest care/support preferences may need to be openly discussed by HCPs with PWD and their caregivers early in the intervention. An associated challenge relates to communication and relationship difficulties that may be experienced by the PWD and caregivers [17], especially as cognitive abilities progressively decline. As well as discussing care/support preferences early in the intervention, communication and relationship difficulties may also need to be addressed. Such potential difficulties will be further explored in the future planned feasibility study. Preferences expressed by caregivers for on-demand guidance will not be possible to incorporate into the adapted intervention delivery model, given intervention session frequency and duration follow current evidence-based BA treatment protocols [21]. Further, evidence suggests that on-demand guidance may be associated with higher levels of dropout [61] and the provision of structured dyadic and individual (caregiver) guidance sessions reduces the burden associated with participating in dyadic interventions for PWD and caregivers [44, 62]. The acceptability and feasibility of the intervention delivery model and provision of regular scheduled guidance will be explored in the next phase of this research.

Finally, some caregivers questioned the feasibility of supporting the PWD use the intervention due to caregiver burden, especially if the PWD required much support to use the intervention. Similar concerns have previously been expressed by caregivers regarding the acceptability and feasibility of other dyadic interventions [56, 59]. Caregiver burden may be a barrier to intervention engagement and delivery, given other informal caregiving demands with burden increasing over time, given the progressive nature of dementia including worsening neuropsychiatric symptoms, function, and overall health [63]. Caregiver burden may therefore be a barrier to intervention engagement and delivery, which will need to be further explored in future research.

### Limitations

The current study has limitations that should be considered. First, data were analyzed with manifest content analysis, striving for a low degree of interpretation. Consequently, there is some overlap between interview guide topics and subcategories (e.g., delivery mode, setting, support and guidance). However, categories deviating too far from the manifest data (i.e., responses to questions in topic guides) may indicate an interpretive analysis inappropriate given the manifest content analysis approach adopted. Second, stakeholder views were elicited from a brief intervention description alongside the workbooks, which may have limited participants’ ability to provide more specific feedback on the intervention. An alternative approach could have been a vignette method whereby recordings of sample guidance sessions are provided to participants to better explore their views of the intervention in practice [64]. However, this study was intended to be exploratory to inform intervention development, and stakeholder views on intervention acceptability and feasibility will be explored in a future feasibility study. Third, due to resource limitations, it was not possible to translate study materials and workbook material into languages other than Swedish e.g., official Swedish minority languages such as Finnish and Sami and other commonly spoken languages in Sweden such as Arabic and Persian. Consequently, speakers of minority languages who are not fluent in Swedish were excluded and findings may not be considered transferable to these groups. This is of particular importance given ethnic minority groups with dementia are at high risk of marginalization given a lack of culturally appropriate services [65] and interventions [66]. However, the intervention adaptation approach used can serve as a model for future intervention adaptations for ethnic minority groups. Fourth, past or present experience of depression was not an inclusion criterion for PWD. No depression symptom screening was conducted and PWD did not need to meet diagnosis for major depression to be included. Therefore, results may not be transferable to a clinically depressed population. However, the majority of included PWD reported current or previous problems with low mood or psychological well-being. Given the LI-BA intervention is designed for PWD with mild-to-moderate symptoms of low mood/depression, those included were likely representative of the target population of the future intervention.

Despite these limitations, we have adopted a structured and systematic approach to intervention development, following the MRC framework [23]. By involving multiple stakeholders, we were able to successfully collect in-depth qualitative data to increase the acceptability and relevancy of the intervention [24, 67]. To strengthen the credibility and confirmability of findings, we used independent coders, held multiple data analysis workshops, conducted peer examination, and applied disconfirming case analysis [42].

## Conclusion

The idea of the proposed LI-BA intervention, including workbook materials and the intervention delivery model, was generally well received by HCPs, and community stakeholders, caregivers, and PWD. We were able to explore needs and preferences to inform adaptation and tailoring of the intervention to enhance cultural appropriateness, relevancy, and acceptability for a currently neglected population. In accordance with the MRC complex interventions framework [23], results will be used to inform a future planned feasibility study to further explore the acceptability and feasibility of the intervention.

### Electronic supplementary material

Below is the link to the electronic supplementary material.

**Additional file 1:** Written summary of intervention delivery model

**Additional file 2:** Fig. S1 Intervention delivery model

**Additional file 3:** Interview topic guides for each stakeholder group

**Additional file 4:** Table S1 Supporting quotations

**Additional file 5:** Standards for Reporting Qualitative Research Checklist

## Data Availability

The datasets generated and/or analyzed during the current study are not publicly available due to privacy or ethical restrictions but are available from the corresponding author on reasonable request.

## Abbreviations

BA: Behavioral activation
CBT: Cognitive behavioral therapy
HCP: Healthcare professional
LI-BA: Low-intensity behavioral activation
LI-CBT: Low-intensity cognitive behavioral therapy
MRC: Medical research council
PWD: People with dementia
