# Supplementary material for: Adaptation of a guided low-intensity behavioral activation intervention for people with dementia in Sweden: a qualitative study exploring the needs and preferences of key stakeholders
Source: BMC Geriatr. 2024 Jan 30;24:113. doi: 10.1186/s12877-023-04606-6 (PMC10826011; doi:10.1186/s12877-023-04606-6)

involvera

UPPSALA UNIVERSITET

INVOLVERA: Development of a support programme to help people with dementia to live well

The description below is a proposal for how the support programme INVOLVERA could be delivered. This is how the programme was delivered in the United Kingdom. Together with you, we want to develop and adapt the programme for the Swedish context. We would like to hear your views regarding what you think would be most suitable in Sweden, e.g., how often people with dementia and their informal caregivers need support, how the support is given, and who should provide the support.

What is INVOLVERA about?

The INVOLVERA programme was originally developed together with people experiencing memory difficulties and their partners, family members, and friends in the United Kingdom.

The programme is designed to help people living with memory difficulties to live well with memory difficulties and improve wellbeing. INVOLVERA is based on a technique called Behavioural Activation and is delivered through two workbooks. One workbook is designed for the person with memory difficulties. The second workbook is designed for a partner, family member, or friend.

The person living with memory difficulties is supported by a partner, family member or friend to work with Behavioural Activation. The partner, family member, or friend receives guidance from a healthcare professional.

The INVOLVERA programme normally lasts for a maximum of 12 weeks.

What is Behavioural Activation?

Behavioural Activation is an evidence-based psychological technique designed to help people to improve their wellbeing.

When people experience memory difficulties they may stop doing many activities they used to do. This might be because some activities are harder due to memory difficulties.

People also stop doing activities when they are experiencing difficulties with their wellbeing, for example, when they are feeling low, fed-up, and sad.

People may feel too tired. Or may not feel bothered to do activities as they do not feel they will enjoy the activities like they used to. Or some tasks may feel much harder to do.

Behavioural Activation helps people with memory difficulties to start to do activities again – a little at a time.

Who is involved in the INVOLVERA programme?

- The person living with memory difficulties.
- A partner, family member, or friend to support the person with memory difficulties.
- A healthcare professional who will provide guidance throughout the programme.

What does the support look like?

Initial sessions

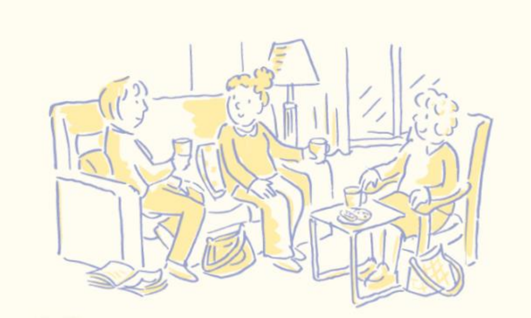

The person living with memory difficulties and the partner, family member or friend has two meetings with the healthcare professional.

At these meetings, the healthcare professional will ask questions to understand a little bit more what kind of problems the person living with memory difficulties is experiencing.

The healthcare professional will also introduce the INVOLVERA programme and the two workbooks.

Between the two meetings, the person living with memory difficulties and the partner, family member or friend will start to work with the INVOLVERA programme and workbooks.

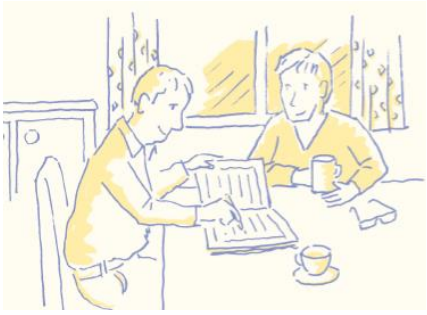

Weekly support sessions

The partner, family member or friend will have weekly support sessions with the healthcare professional over telephone. The support sessions are designed to help the partner, family member, or friend overcome any obstacles they might be experiencing using the INVOLVERA programme and discuss the next steps.

Final session

The final session of the programme is a meeting where the person living with memory difficulties and the partner, family member, or friend, meet with the healthcare professional.

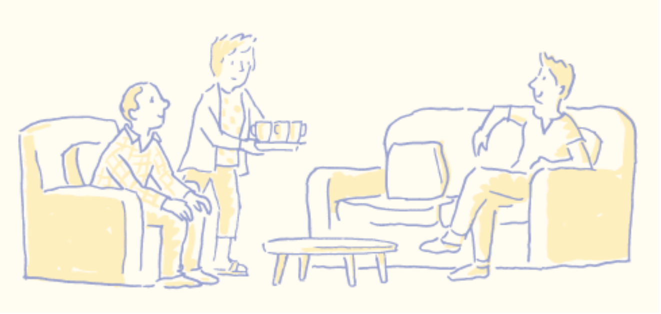

During this meeting, the healthcare professional will help the person living with memory difficulties and the partner, family member, or friend learn how to continue to use techniques from the programme in the future.

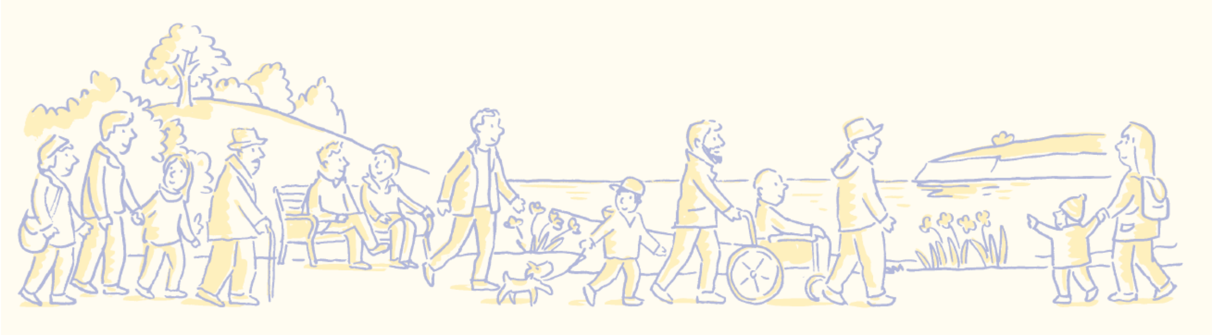

involvera

The Support Programme INVOLVERA

UPPSALA UNIVERSITET

INVOLVERA: Developing a support programme to help people living with memory difficulties to live well

- **The description below is a proposal for how the support programme INVOLVERA could be delivered**
- **This is how the programme was delivered in the United Kingdom**
- **Together with you, we want to develop and adapt the programme for Sweden**
- **We would like to hear your views regarding what you think would be most suitable in Sweden**

What is the programme INVOLVERA about?

INVOLVERA was developed together with people experienceing memory difficulties and their partners, family members, and friends in the United Kingdom.

The support programme is designed to help people living with memory difficulties to live well with memory difficulties and improve wellbeing.

The support programme is based on a technique called *Behavioural Activation* and is delivered through two workbooks. One workbook is designed for the person with memory difficulties. The second workbook is designed for a partner, family member, or friend.

The person living with memory difficulties is supported by a partner, family member, or friend to work with *Behavioural Activation.* The partner, family member, or friend receives guidance from a healthcare professional.

The support programme normally lasts for 12 weeks.

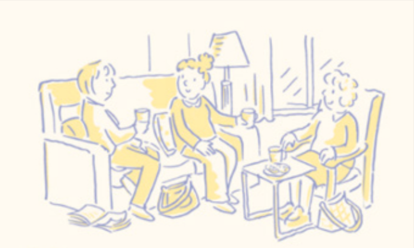

involvera

UPPSALA UNIVERSITET

What is Behavioural Activation?

Behavioural Activation is a technique designed to help people to improve their wellbeing.

When people experience memory difficulties they may stop doing many activities they used to do.

This might be because some activities are harder due to memory difficulties, also people may feel low, and sad, or too tired.

Behavioural Activation helps people with memory difficulties to start to do activities again – a little at a time.

Who is involved in INVOLVERA?

- The person living with memory difficulties.
- A partner, family member, or friend to support the person with memory impairment.
- A guidance person from healthcare who will provide guidance throughout the programme.

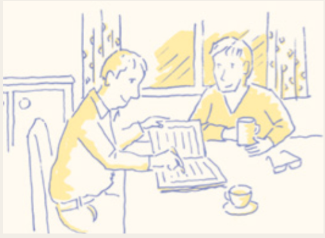

What does the support look like?

Two initial sessions

- The person living with memory difficulties and the partner, family member, or friend has two meetings with the guidance person.
- At the first meeting the guidance person will ask questions to understand a little bit more what kind of problems the person living with memory difficulties is experiencing. The guidance person will also introduce the workbooks.
- At the second meeting (one and a half weeks after first meeting) the guidance person will explain how the INVOLVERA programme works and the purpose of the programme

involvera

UPPSALA
UNIVERSITET

Weekly support sessions

- The partner, family member, or friend will have weekly support sessions with the guidance person over telephone
- The support sessions are designed to help the partner, family member, or friend overcome any obstacles they might be experiencing using the programme and discuss the next steps.

Final session

- The final session is a meeting where the person living with memory difficulties and the partner, family member, or friend, meet with the guidance person
- During this meeting, the guidance person will help the person living with memory difficulties and the partner, family member, or friend learn how to continue to use techniques from the programme in the future

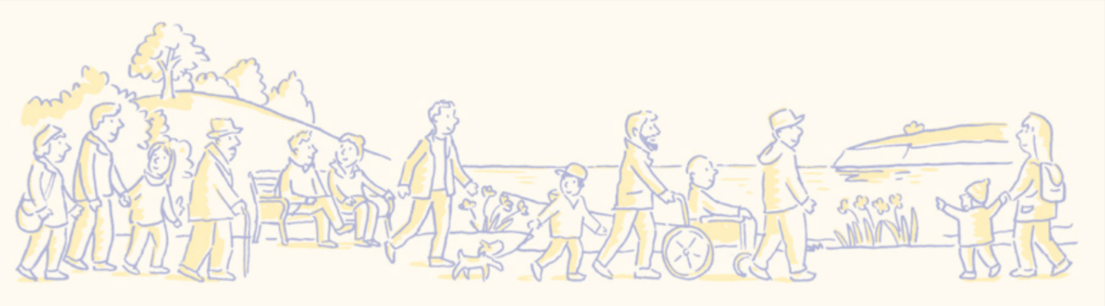

Supplement: Supplementary file 1 — Additional file 1: Written summary of intervention delivery model [file 12877_2023_4606_MOESM1_ESM.pdf]
