# Supplementary material for: Adaptation of a guided low-intensity behavioral activation intervention for people with dementia in Sweden: a qualitative study exploring the needs and preferences of key stakeholders
Source: BMC Geriatr. 2024 Jan 30;24:113. doi: 10.1186/s12877-023-04606-6 (PMC10826011; doi:10.1186/s12877-023-04606-6)
Supplement: Supplementary file 2 — Additional file 2: Fig. S1 Intervention delivery model [file 12877_2023_4606_MOESM2_ESM.docx]

Copyright © 2021, BMJ Publishing Group Ltd. All rights reserved.

**Fig. S1** Intervention delivery model (Previously published in: Svedin et al., 2021 [29])
