# Supplementary material for: Adaptation of a guided low-intensity behavioral activation intervention for people with dementia in Sweden: a qualitative study exploring the needs and preferences of key stakeholders
Source: BMC Geriatr. 2024 Jan 30;24:113. doi: 10.1186/s12877-023-04606-6 (PMC10826011; doi:10.1186/s12877-023-04606-6)
Supplement: Supplementary file 3 — Additional file 3: Interview topic guides for each stakeholder group [file 12877_2023_4606_MOESM3_ESM.docx]

**Additional file 3** Interview topic guides for each stakeholder group

**Healthcare professionals and Community stakeholders**

1. How would you describe INVOLVERA?
2. What do you think is the purpose of INVOLVERA?
3. What are your first impressions of INVOLVERA?
4. The workbooks state that a person from U-CARE will guide the informal caregiver (this is only an example). At present, there is no identified (professional) group that can do this. Who do you think is best able to provide guidance to informal caregivers? [prompt: dementia care consultants, nurses, psychologists, volunteers, others]
5. There are different approaches to providing guidance. For example, it can be done over the phone, via a physical meeting, via email, or in a group setting. What approach do you think would be best to provide this guidance? [Explore answers]
6. If guidance is given through a physical meeting, where do you think it should take place? [prompts: home, doctor's surgery, memory clinic, daycare, other]
7. How often do you think informal caregivers would need guidance in using the workbook?

Questions about the workbooks:

1. What are your impressions of the workbooks?
2. What did you like and what did you not like?
3. How relevant do you think the workbook is for people with dementia?
4. How relevant do you think the workbook is for informal caregivers?
5. How do you think the workbooks can be improved?
6. On a page in the workbook for informal caregivers, we will add a list of different sources of caregiver support - do you have any suggestions on support organizations / groups / places for informal caregivers?

**People with dementia**

1. How would you describe INVOLVERA?

a) What do you think is the purpose of INVOLVERA?

1. What are your first impressions of INVOLVERA?
2. Who do you think is best able to provide guidance to informal caregivers? [prompt: dementia care consultants, nurses, psychologists, volunteers, others]
3. There are different approaches to providing guidance. For example, it can be done over the phone, via a physical meeting, via email, or in a group setting. What approach do you think would be best to provide this guidance?
4. Which approach would you prefer?
5. If guidance is given through a physical meeting, where do you think it should take place? [prompts: home, doctor's office, memory clinic, daycare, other]
6. How often do you think people with memory difficulties would need support in using the workbook?
7. How often would you need support in using the workbook?

Questions about the workbooks:

1. What are your impressions of the workbooks?
2. What did you like and what did you not like?
3. How relevant do you think the workbook is for people with memory difficulties?
4. How relevant do you think the workbook is to you?
5. How do you think the workbooks can be improved?

[prompts: language (word choice, sentence structure, readability, content, use of metaphors and similes), illustrations (appearance, activities, relevance), examples (names, scenarios, activities, relevance).

Word choice examples: memory difficulties/impairment, dementia, cognitive impairment, other?

1. The workbooks are now provided in a written format. Is there any other delivery format you would prefer? [prompt: online, audiobook, via computer, tablet, mobile application]

**Informal caregivers**

1. How would you describe INVOLVERA?

a) What do you think is the purpose of INVOLVERA?

1. What are your first impressions of INVOLVERA?
2. Who do you think is best able to provide guidance to informal caregivers? [prompt: dementia care consultants, nurses, psychologists, volunteers, others]
3. There are different approaches to providing guidance. For example, it can be done over the phone, via a physical meeting, via email, or in a group. What approach do you think would be best to provide this guidance?
4. Which approach would you prefer?
5. If guidance is given through a physical meeting, where do you think it should take place? [prompts: home, doctor's office, memory clinic, daycare, other]
6. How often do you think informal caregivers would need guidance in using the workbook?
7. How often would you need guidance in using the workbook?

Questions about the workbooks:

1. What are your impressions of the workbooks?
2. What did you like and what did you not like?
3. How relevant do you think the workbook is for people with memory difficulties?
4. How relevant do you think the workbook is to informal caregivers?
5. How relevant do you think the workbook is to you?
6. How do you think the workbooks can be improved?

[prompts: language (word choice, sentence structure, readability, content, use of metaphors and similes), illustrations (appearance, activities, relevance), examples (names, scenarios, activities, relevance). Word choice examples: memory difficulties/impairment, dementia, cognitive impairment, other?]

1. On a page in the workbook for informal caregivers, we will add a list of different sources of caregiver support - do you have any suggestions on support organizations / groups / places for informal caregivers?
2. The workbooks are now provided in a written format. Is there any other delivery format you would prefer? [prompt: online, audiobook, via computer, tablet, mobile application]
