# Supplementary material for: Adaptation of a guided low-intensity behavioral activation intervention for people with dementia in Sweden: a qualitative study exploring the needs and preferences of key stakeholders
Source: BMC Geriatr. 2024 Jan 30;24:113. doi: 10.1186/s12877-023-04606-6 (PMC10826011; doi:10.1186/s12877-023-04606-6)
Supplement: Supplementary file 4 — Additional file 4: Table S1 Supporting quotations [file 12877_2023_4606_MOESM4_ESM.docx]

**Table S1** Supporting quotations

| **Categories and subcategories** | **Stakeholder** | **Quotations†** |
| --- | --- | --- |
|  |  |  |
| Content |  |  |
| - Cultural aspects | HCP 2 | A very silly thing but it said “to put on a cup of tea” [in the workbook]. In Sweden, you would probably write that you “start the coffee maker”, maybe “make a cup of coffee”. |
|  | PWD 5 | [Take] a walk in the forest … I love being out in nature. |
|  | Caregiver 18 | For all people... it [life situation] is so different, you live differently and have different friendships... and everything like that |
|  |  |  |
| - Inclusivity |  |  |
|  | Community stakeholder 7 | People who are younger than 65 and who have received a cognitive impairment diagnosis, they are incredibly sensitive... You must not be derogatory in any way. |
|  |  |  |
|  | Caregiver 11 | Memory impairment [as a term] for dementia is good. A lot of people react and think it [dementia] is embarrassing or something. I think memory impairment is good. |
|  | PWD 3 | A lot of it [the illustrations] is old grandmothers and old aunts in wheelchairs. You do not have to look old and scrawny to have it [dementia], and you think yourself that you are still 25 [years old]. |
|  | Caregiver 12 (Disconfirming case)  Caregiver 16  (Disconfirming case) | Memory impairment sounds a little mean so to speak. I think dementia is a more sensible word.  It [depression] is a clinically important diagnosis. You need to define what you mean by depression. Because there are many people who misunderstand this with depression, and if you are a little depressed, you talk about depression, depressive symptoms, and so on. It is nothing compared to a depression, a clinical depression. |
| Delivery procedures |  |  |
| - Availability | HCP 6  HCP 1 | I also believe that you should have both something [guidance sessions] planned and organized once a week and then so that you can also turn to someone [guidance person] when needed and that it is easy to get hold of that person.  Some people will need guidance every time they open it [the workbook] because they are so unsure of how to use it, and others will understand them immediately... I think it is very individual. |
|  | Community stakeholder 7  Community stakeholder 3 | First of all, there should be the possibility to sound the alarm [contact guidance person] at all times, and that there is a telephone number that you could call and be called on.  But I think that is a pretty good amount of time there [guidance sessions]. Because it [guidance sessions] must not be too often, because then I think you feel coerced to do it. I think once a week would be good. |
|  | PWD 3 | It can be good to have [guidance sessions] at a regular interval to know when these meetings are, both for the person who is ill, even if you might forget from time to time. But above all for the caregivers, to know that in 14 days there is a meeting. Then we can talk again and try to discuss this [problem] that has come up now and how to solve it. |
|  | Caregiver 13  Caregiver 4 | It [guidance] is great on the telephone, if you know where to call. Because things [problems working with intervention] can pop up very quickly.  Once a week is a good idea because it is enough time to do something in between [guidance sessions], but it is also short enough to not lose momentum and forget. |
|  |  |  |
| - Delivery mode | HCP 9 | The physical meeting [is best for guidance sessions]. I think you need a few [physical meetings] in the beginning to build up some kind of trust |
|  | Community stakeholder 3 | Once a week you could just [have telephone guidance] ... The conversation may not need to last more than a few minutes… You give advice and then they agree to try it … if it does not work then they will bring this up at our physical meeting and see if we can help. |
|  | PWD 8 | Yes [prefer workbook in paper format], I do not think I would want one that was on gramophone [audiobook]. |
|  | Caregiver 4 | The telephone is simple. You can be almost anywhere... It takes less time compared to going somewhere. You can sit in the car if that is the case. |
|  | Community stakeholder 3  (Disconfirming case) | I do not believe in telephone contact. I am a little worried about that… because you can never have the same kind of communication with each other if you do not see each other. |
| - Setting | HCP 17 | I think that if you don’t meet at home, the conversation can be freer if the person who is sick is not there. It is a little more restrained if it is at home, so a neutral place [is better]. |
|  | Community stakeholder 5 | Then it [guidance sessions] is probably better to be at home in a calm and safe environment with the family or if you do not have such a big family but some good friends or something that you can feel safe with. |
|  | PWD 6 | It might be good that it [guidance sessions] are not in a hospital environment. |
|  |  |  |
| - Support and guidance | HCP 14 | I also think that caregivers themselves may need a lot of support to cope. And it is also written in the books here that you need it. The step to getting help can be very long sometimes. They [caregivers] need a lot of guidance. |
|  |  |  |
|  | Caregiver 17 | It would be necessary with one more [physical guidance session] for the caregiver when you have got into it [the intervention] a bit. Then have these [weekly] telephone sessions… You would probably need a booster [session] eventually. |
|  |  |  |
| Illness trajectory |  |  |
| - Burden of care | HCP 6 | In the worst case, it [supporting the intervention] will be a pressure on relatives who feel that it is their responsibility now to ensure that the patient or the person who has dementia does all these things. If you then have a person who has difficulty getting things started or has apathy, there is a risk of [the intervention] placing demands and pressure and [feelings of] failure for relatives or those who are supposed to help [support the intervention]. |
|  | Community stakeholder 2 | If it [the intervention] becomes too academic, too complex, and comprehensive, then I think that you will get tired of it. |
| - Burden of material | HCP 11 | The material [workbooks] is large [a lot of text]. If you get this compendium, it can feel overwhelming, I can imagine. |
|  | Community stakeholder 2 | Well, I have probably said this a lot of times here, that it [workbooks] can feel a bit heavy [with a lot of text]. |
|  | PWD 6 | There was a lot of text [in the workbook]. |
|  | Caregiver 5 | There is quite a lot [of text] in them [workbooks]. I am still reasonably used to reading, so the question is whether everyone [PWD and caregivers] will understand or have the stamina to read. |
| - Timing | HCP 16 | It is [important] that you get a handle on the people [with dementia] early so that you can come in with the support early in the illness... as early as possible, then I think you have great possibilities. |
|  |  |  |
|  | Caregiver 10 | It [the intervention] should start already when it [memory impairment] is suspected. |

† Quotations were translated from Swedish to English by one author (Swedish first language, English second language) and one author (English first language, some knowledge in Swedish) who provided feedback on English wording, and language nuances, and confirmed that translated quotations were understandable. To protect the identity of stakeholders, names, specific locations, and other personal information have been replaced with generic information in squared brackets if needed.
